# Supplementary material for: Food insecurity and mental health among migrants and refugees in high-income countries: Systematic review and meta-analyses
Source: PLoS One. 2026 Feb 18;21(2):e0342128. doi: 10.1371/journal.pone.0342128 (PMC12915952; doi:10.1371/journal.pone.0342128)
Supplement: S1 File — (DOCX) [file pone.0342128.s001.docx]

S1 File. Protocol: Published protocol on Food insecurity and psychological stress among migrants and refugees in high-income countries.
