# Supplementary material for: Food insecurity and mental health among migrants and refugees in high-income countries: Systematic review and meta-analyses
Source: PLoS One. 2026 Feb 18;21(2):e0342128. doi: 10.1371/journal.pone.0342128 (PMC12915952; doi:10.1371/journal.pone.0342128)
Supplement: S1 Appendix — (DOCX) [file pone.0342128.s004.docx]

S1 Appendix. Full database search strategies.

**Table 4- Medline Search**

| **Database** | **Search terms** | **Items found** |
| --- | --- | --- |
| **Food insecurity and mental stress** | | |
| **#1** | "Transients and Migrants"/ | **14578** |
| **#2** | exp "Emigrants and Immigrants"/ | **15947** |
| **#3** | Refugees/ | **13570** |
| **#4** | (migrant* or immigrant* or emigrant* or asylum seeker* or refugee* or displaced person* or undocumented migrant*).mp. | **77755** |
| **#5** | 1 or 2 or 3 or 4 | **77755** |
| **Another search** | | |
| **#6** | stress, psychological/ or burnout, psychological/ or financial stress/ | **137930** |
| **#7** | Anxiety/ | **110614** |
| **#8** | Depression/ | **155146** |
| **#9** | Mental Health/ | **65284** |
| **#10** | Psychological Distress/ | **4423** |
| **#11** | Financial Stress/ | **1192** |
| **#12** | stress, psychological/ or burnout, psychological/ or financial stress/ | **137930** |
| **#13** | (psychological stress* or psychological distress* or burn out* or anxiety* or depression* or mental health* or financial stress* or psychological burn out*).mp. | **927896** |
| **#14** | 6 or 7 or 8 or 9 or 10 or 11 or 12 or 13 | **1010896** |
| **Another Search** | | |
| **#15** | exp food insecurity/ or access to healthy foods/ | **1620** |
| **#16** | exp food security/ or access to healthy foods/ | **770** |
| **#17** | Hunger/ | **6114** |
| **#18** | Food Deprivation/ | **8739** |
| **#19** | (Food insecurity* or hunger* or food deprivation* or food poverty* or food scarcity* or inadequate food access* or food security* or food crisis*).mp. | **44733** |
| **#20** | 15 or 16 or 17 or 18 or 19 | **44752** |
| **Another Search** | | |
| **#21** | (America* or Andorra* or Antigua* or Aruba* or Australia* or Austria* or Barbuda* or Bermuda* or Britain or British or Baham* or Bahrain* or Barbad* or Belgium or Belgian* or Brunei* or Canada or Canadian* or Cayman Island* or Channel Island* or Chile* or Croatia* or Curacao* or Cyprus or Cyprian* or Cypriot? or Czech* or Darussalam or Denmark or Danish or England or English or Estonia* or Faroe Island* or Finland or Finnish or Finn? or France or French or German* or Gibralta* or Greece or Greek* or Greenland* or Guam* or Hong Kong* or Hungary or Hungarian* or Iceland* or Ireland or Irish or "Isle of Man" or Israel* or Italy or Italian* or Japan* or South Korea* or Kuwait* or Latvia* or Liechtenstein* or Lithuania* or Luxembourg* or Macao* or Malta or Maltese or Monaco or Nauru* or Netherlands or Dutch or New Caledonia* or New Zealand* or Northern Mariana Island* or Norway or Norwegian* or Oman* or Panama* or Poland or Polish or Portug* or Puerto Ric* or Romania* or Qatar* or Saint Kitts or San Marino or Saint Martin or Sint Maarten or Saudi Arabia* or Seychelles or Singapore* or Slovak* or Slovenia* or Spain or Spanish or Sweden or Swedish or Switzerland or Swiss or Taiwan* or Trinidad* or Tobago* or (Turks and Caicos Island*) or United Arab Emirates or United Kingdom or UK or United States or USA or Uruguay* or Virgin Island* or (western adj (country* or economy* or nation*))).mp. | **5958671** |
| **#22** | (America* or Andorra* or Antigua* or Aruba* or Australia* or Austria* or Barbuda* or Bermuda* or Britain or British or Baham* or Bahrain* or Barbad* or Belgium or Belgian* or Brunei* or Canada or Canadian* or Cayman Island* or Channel Island* or Chile* or Croatia* or Curacao* or Cyprus or Cyprian* or Cypriot? or Czech* or Darussalam or Denmark or Danish or England or English or Estonia* or Faroe Island* or Finland or Finnish or Finn? or France or French or German* or Gibralta* or Greece or Greek* or Greenland* or Guam* or Hong Kong* or Hungary or Hungarian* or Iceland* or Ireland or Irish or "Isle of Man" or Israel* or Italy or Italian* or Japan* or South Korea* or Kuwait* or Latvia* or Liechtenstein* or Lithuania* or Luxembourg* or Macao* or Malta or Maltese or Monaco or Nauru* or Netherlands or Dutch or New Caledonia* or New Zealand* or Northern Mariana Island* or Norway or Norwegian* or Oman* or Panama* or Poland or Polish or Portug* or Puerto Ric* or Romania* or Qatar* or Saint Kitts or San Marino or Saint Martin or Sint Maarten or Saudi Arabia* or Seychelles or Singapore* or Slovak* or Slovenia* or Spain or Spanish or Sweden or Swedish or Switzerland or Swiss or Taiwan* or Trinidad* or Tobago* or (Turks and Caicos Island*) or United Arab Emirates or United Kingdom or UK or United States or USA or Uruguay* or Virgin Island* or (western adj (countr* or econom* or nation*))).sh. | **2831148** |
| **#23** | ((high* or upper) adj5 income? adj5 (countr* or econom* or group? or nation?)).mp. | **19856** |
| **#24** | 21 or 22 or 23 | **5971068** |
| **#25** | 5 and 14 and 20 and 24 | **79** |
|  |  |  |

**Table 5 -Web of Science search strategy**

| **Database** | **Search terms** | **Items found** |
| --- | --- | --- |
| **Food insecurity and mental stress** | | |
| **#1** | ALL=('Transients' or 'migrant' or 'immigrant 'or 'emigrant' or 'asylum seeker' or 'refugee' or 'displaced person' or 'undocumented migrant') | Results: 811773 |
| **Another search** | | |
| **#2** | ALL=('Anxiety' or 'Depression' or 'Mental Health' or 'Psychological Distress' or 'Psychological stress' or 'Financial Stress' or 'burn out' or 'Psychological burn out') | Results: 1581997 |
| **Another Search** | | |
| **#3** | ALL=('Food insecurity' or 'hunger' or 'food deprivation' or 'food poverty' or 'food scarcity' or 'inadequate food access' or 'food security' or 'food crisis') | Results: 144280 |
| **Another Search** | | |
| **#4** | TS=(America* or Andorra* or Antigua* or Aruba* or Australia* or Austria* or Barbuda* or Bermuda* or Britain or British or Baham* or Bahrain* or Barbad* or Belgium or Belgian* or Brunei* or Canada or Canadian* or Cayman Island* or Channel Island* or Chile* or Croatia* or Curacao* or Cyprus or Cyprian* or Cypriot or Czech* or Darussalam or Denmark or Danish or England or English or Estonia* or Faroe Island* or Finland or Finnish or Finn or France or French or German* or Gibralta* or Greece or Greek* or Greenland* or Guam* or Hong Kong* or Hungary or Hungarian* or Iceland* or Ireland or Irish or "Isle of Man" or Israel* or Italy or Italian* or Japan* or South Korea* or Kuwait* or Latvia* or Liechtenstein* or Lithuania* or Luxembourg* or Macao* or Malta or Maltese or Monaco or Nauru* or Netherlands or Dutch or New Caledonia* or New Zealand* or Northern Mariana Island* or Norway or Norwegian* or Oman* or Panama* or Poland or Polish or Portug* or Puerto Ric* or Romania* or Qatar* or Saint Kitts or San Marino or Saint Martin or Sint Maarten or Saudi Arabia* or Seychelles or Singapore* or Slovak* or Slovenia* or Spain or Spanish or Sweden or Swedish or Switzerland or Swiss or Taiwan* or Trinidad* or Tobago* or Turks and Caicos Island* or United Arab Emirates or United Kingdom or UK or United States or USA or Uruguay* or Virgin Island* or western adj countr* or econom* or nation*) | Results: 12058946 |
| **#5** | #1 AND #2 AND #3 AND #4 | Results: 193 |
|  |  |  |

**Table 6 -EMBASE search strategy**

| **Database** | **Search terms** | **Items found** |
| --- | --- | --- |
| **Food insecurity and mental stress** | | |
| **#1** | (Transients or migrant or immigrant or emigrant or asylum seeker or refugee or displaced person or undocumented migrant).af. | Results: 91609 |
| **Another search** | | |
| **#2** | (Anxiety or Depression or Mental Health or Psychological Distress or Psychological stress or Financial Stress or burn out or Psychological burn out).af. | Results: 1658717 |
| **Another Search** | | |
| **#3** | (Food insecurity or access to healthy foods or food security or hunger or food deprivation or food poverty or food scarcity or inadequate food access or food crisis).af. | Results: 57,174 |
| **Another Search** | | |
| **#4** | (America or Andorra or Antigua or Aruba or Australia or Austria or Barbuda or Bermuda or Britain or British or Baham or Bahrain or Barbad or Belgium or Belgian or Brunei or Canada or Canadian or Cayman Island or Channel Island or Chile or Croatia or Curacao or Cyprus or Cyprian or Cypriot or Czech or Darussalam or Denmark or Danish or England or English or Estonia or Faroe Island or Finland or Finnish or Finn or France or French or German or Gibralta or Greece or Greek or Greenland or Guam or Hong Kong or Hungary or Hungarian or Iceland or Ireland or Irish or Isle of Man or Israel or Italy or Italian or Japan or South Korea or Kuwait or Latvia or Liechtenstein or Lithuania or Luxembourg or Macao or Malta or Maltese or Monaco or Nauru or Netherlands or Dutch or New Caledonia or New Zealand or Northern Mariana Island or Norway or Norwegian or Oman or Panama or Poland or Polish or Portug or Portugal or Puertoric or Romania or Qatar or Saint Kitts or Sanmarino or Saint Martin or Sint Maarten or Saudi Arabia or Seychelles or Singapore or Slovak or Slovenia or Spain or Spanish or Sweden or Swedish or Switzerland or Swiss or Taiwan or Trinidad or Tobago or Turks or Caicos Island or United Arab Emirates or United Kingdom or UK or United States or USA or Uruguay or Virgin island or Western country or economy or nation or high income country or economy or nation).af. | Results: 43,635,973 |
| **#5** | #1 AND #2 AND #3 AND #4 | Results: 214 |
|  |  |  |

**Table 7: PsycINFO search strategy**

| **Database** | **Search terms** | **Items found** |
| --- | --- | --- |
| **Food insecurity and mental stress** | | |
| **#1** | "transients AND migrants OR emigrants AND immigrants OR refugees OR migrant OR immigrant OR emigrant OR asylum seeker OR displaced person OR undocumented migrant OR refugee" | Results: 55912 |
| **Another search** | | |
| **#6** | "psychological AND stress OR psychological distress OR burn out OR anxiety OR depression OR mental health OR financial stress OR psychological burn out" | Results: 1280723 |
| **Another Search** | | |
| **#15** | "food insecurity OR access to healthy foods OR food security OR hunger OR food deprivation OR food poverty OR food scarcity OR inadequate food access OR food crisis" | Results: 17338 |
| **Another Search** | | |
| **#21** | "(America OR Andorra OR Antigua OR Aruba OR Australia OR Austria OR Barbuda OR Bermuda OR Britain OR British OR Baham OR Bahrain OR Barbad OR Belgium OR Belgian OR Brunei OR Canada OR Canadian OR Cayman Island OR Channel Island OR Chile OR Croatia OR Curacao OR Cyprus OR Cyprian OR Cypriot OR Czech OR Darussalam OR Denmark OR Danish OR England OR English OR Estonia OR Faroe Island OR Finland OR Finnish OR Finn OR France OR French OR German OR Gibralta OR Greece OR Greek OR Greenland OR Guam OR Hong Kong OR Hungary OR Hungarian OR Iceland OR Ireland OR Irish OR Isle of Man OR Israel OR Italy OR Italian OR Japan OR South Korea OR Kuwait OR Latvia OR Liechtenstein OR Lithuania OR Luxembourg OR Macao OR Malta OR Maltese OR Monaco OR Nauru OR Netherlands OR Dutch OR New Caledonia OR New Zealand OR Northern Mariana Island OR Norway OR Norwegian OR Oman OR Panama OR Poland OR Polish OR Portug OR Portugal OR Puertoric OR Romania OR Qatar OR Saint Kitts OR Sanmarino OR Saint Martin OR Sint Maarten OR Saudi Arabia OR Seychelles OR Singapore OR Slovak OR Slovenia OR Spain OR Spanish OR Sweden OR Swedish OR Switzerland OR Swiss OR Taiwan OR Trinidad OR Tobago OR Turks or Caicos Island OR United Arab Emirates OR United Kingdom OR UK OR United States OR USA OR Uruguay OR Virgin island OR (Western adj (country* or econom* or nation OR ( high income adj (country OR economy OR nation)) " | Results: 5,303.073 |
| **#22** | S1 AND S2 AND S3 AND S4 | Results: 144 |
|  |  |  |

**Table 8: New SCOPES**

| **Database** | **Search terms** | **Items found** |
| --- | --- | --- |
| **Migrants and Refugees** | | |
| **#1** | ALL ( transients AND migrants ) | **19416** |
| **#2** | ALL ( emigrants AND immigrants ) | **42284** |
| **#3** | ALL ( refugees ) | **258706** |
| **#4** | ALL ( ( migrant* OR immigrant* OR emigrant* OR asylum AND seeker* OR refugee* OR displaced AND person* OR undocumented AND migrant* ) ) ) | [261,962 results](https://www.scopus.com/search/history/results.uri?origin=searchhistory&shid=5) |
| **#5** | 1 or 2 or 3 or 4 | **261962** |
| **Psychological Stress** | | |
| **#6** | ALL ( stress, AND psychological OR burnout, AND psychological OR financial AND stress ) | [1,146,775 results](https://www.scopus.com/search/history/results.uri?origin=searchhistory&shid=13) |
| **#7** | ALL ( anxiety ) | [2,147,612 results](https://www.scopus.com/search/history/results.uri?origin=searchhistory&shid=45) |
| **#8** | ALL ( depression ) | [3,379,520 results](https://www.scopus.com/search/history/results.uri?origin=searchhistory&shid=46) |
| **#9** | ALL ( mental AND health ) | [4,538,616 results](https://www.scopus.com/search/history/results.uri?origin=searchhistory&shid=47) |
| **#10** | ALL ( psychological AND distress ) | [4,588,669 results](https://www.scopus.com/search/history/results.uri?origin=searchhistory&shid=23) |
| **#11** | ALL ( financial AND stress ) | [4,640,834 results](https://www.scopus.com/search/history/results.uri?origin=searchhistory&shid=48) |
| **#12** | ALL ( psychological AND stress OR psychological AND distress OR burn AND out OR anxiety OR depression OR mental AND health OR financial AND stress OR psychological AND burn AND out ) | [4,647,438 results](https://www.scopus.com/search/history/results.uri?origin=searchhistory&shid=50) |
| **#13** | 6 or 7 or 8 or 9 or 10 or 11 or 12 | [4,647,438 results](https://www.scopus.com/search/history/results.uri?origin=searchhistory&shid=50) |
| **Food Insecurity** | | |
| **#14** | ALL(food insecurity or access to healthy foods) | [76,803 results](https://www.scopus.com/search/history/results.uri?origin=searchhistory&shid=31) |
| **#15** | ALL(food insecurity or access to healthy foods) | [107,130 results](https://www.scopus.com/search/history/results.uri?origin=searchhistory&shid=12) |
| **#16** | ALL ( hunger ) | [323,728 results](https://www.scopus.com/search/history/results.uri?origin=searchhistory&shid=13) |
| **#17** | ALL ( food AND deprivation ) | [439,015 results](https://www.scopus.com/search/history/results.uri?origin=searchhistory&shid=14) |
| **#18** | ALL ( food AND deprivation ) OR ALL ( food AND insecurity OR hunger OR food AND deprivation OR food AND poverty OR food AND scarcity OR inadequate AND food AND access OR food AND security OR food AND crisis ) ) | [452,861 results](https://www.scopus.com/search/history/results.uri?origin=searchhistory&shid=53) |
| **#19** | 14 or 15 or 16 or 17 or 18 | [452,861 results](https://www.scopus.com/search/history/results.uri?origin=searchhistory&shid=53) |
| **High Income countries** | | |
| **#20** | ( ALL ( america ) OR ALL ( andorra ) OR ALL ( antigua ) OR ALL ( aruba ) OR ALL ( australia ) OR ALL ( austria ) OR ALL ( barbuda ) OR ALL ( bermuda ) OR ALL ( britain ) OR ALL ( british ) OR ALL ( baham ) OR ALL ( bahrain ) OR ALL ( barbad ) OR ALL ( belgium ) OR ALL ( belgian ) OR ALL ( brunei ) OR ALL ( canada ) OR ALL ( canadian ) OR ALL ( cayman AND island ) OR ALL ( channel AND island ) OR ALL ( chile ) OR ALL ( croatia ) OR ALL ( curacao ) OR ALL ( cyprus ) OR ALL ( cyprian ) OR ALL ( cypriot OR czech* OR darussalam ) AND ALL ( denmark OR danish OR england OR english OR estonia* OR faroe AND island* OR finland OR finnish OR finn OR france OR french OR german* OR gibralta* OR greece OR greek* OR greenland* OR guam* OR hong AND kong* OR hungary OR hungarian* ) OR ALL ( iceland* OR ireland OR irish OR "Isle of Man" OR israel* OR italy OR italian* OR japan* OR south AND korea* OR kuwait* OR latvia* OR liechtenstein* OR lithuania* OR luxembourg* OR macao* OR malta OR maltese OR monaco OR nauru* OR netherlands OR dutch OR new AND caledonia* OR new AND zealand* OR northern AND mariana AND island* OR norway OR norwegian* OR oman* OR panama* ) OR ALL ( poland OR polish OR portug* OR portugal ) OR ALL ( puerto AND ric* OR romania* OR qatar* OR saint AND kitts OR san AND marino OR saint AND martin OR sint AND maarten OR saudi AND arabia* OR seychelles OR singapore* OR slovak* OR slovenia* OR spain OR spanish ) OR ALL ( sweden OR swedish OR switzerland OR swiss OR taiwan* OR trinidad* OR tobago* ) OR ALL ( ( turks AND caicos AND island* ) OR united AND arab AND emirates OR united AND kingdom OR uk OR united AND states OR usa OR uruguay* OR virgin AND island* ) OR ALL ( western AND country* OR economy* OR nation* ) OR ALL ( high AND income AND country* OR economy* OR nation* ) ) | [7,535,908 results](https://www.scopus.com/search/history/results.uri?origin=searchhistory&shid=37) |
| **#21** | 5 and 13 and 19 and 20 | [128 results](https://www.scopus.com/search/history/results.uri?origin=searchhistory&shid=27) |
|  |  |  |
