# Supplementary material for: Food insecurity and mental health among migrants and refugees in high-income countries: Systematic review and meta-analyses
Source: PLoS One. 2026 Feb 18;21(2):e0342128. doi: 10.1371/journal.pone.0342128 (PMC12915952; doi:10.1371/journal.pone.0342128)
Supplement: S1 Table — (DOCX) [file pone.0342128.s005.docx]

| S1 Table. Study-level methodological appraisal (Joanna Briggs Institute checklist) for included studies | | | | | | | | | | | | | | | | | | | | | | |
| --- | --- | --- | --- | --- | --- | --- | --- | --- | --- | --- | --- | --- | --- | --- | --- | --- | --- | --- | --- | --- | --- | --- |
| Research Article | 1. Were the criteria for inclusion in the sample clearly defined? | | 2. Were the study subjects and the setting described in detail? | | 3. Was the exposure measured validly and reliably? | | 4. Were objective, standard criteria used for measurement of the condition? | | 5. Were confounding factors identified? | | 6. Were strategies to deal with confounding factors stated? | | 7. Were the outcomes measured validly and reliably? | | 8. Was appropriate statistical analysis used? | | Quality Score % | | | Overall Appraisal | | |
|  | Yes | No | Yes | No | Yes | No | Yes | No | Yes | No | Yes | No | Yes | No | Yes | No | No |  |  | |  |  |
| Zangiabadi et al. (2024) | 1 | 0 | 1 | 0 | 1 | 0 | 1 | 0 | 1 | 0 | 1 | 0 | 1 | 0 | 1 | 0 | 0 | 9 | Low Risk | | Included |  |
| Sonia Lai (2020). | 1 | 0 | 1 | 0 | 1 | 0 | 1 | 0 | 1 | 0 | 1 | 0 | 1 | 0 | 1 | 0 | 0 | 9 | Low Risk | | Included |  |
| Hadley et al. (2008). | 1 | 0 | 1 | 0 | 1 | 0 | 1 | 0 | 1 | 0 | 1 | 0 | 1 | 0 | 1 | 0 | 0 | 9 | Low Risk | | Included |  |
| Patel et al. (2022) | 1 | 0 | 1 | 0 | 1 | 0 | 1 | 0 | 1 | 0 | 1 | 0 | 1 | 0 | 1 | 0 | 0 | 9 | Low Risk | | Included |  |
| Talham (2023) | 1 | 0 | 1 | 0 | 1 | 0 | 1 | 0 | 1 | 0 | 1 | 0 | 1 | 0 | 1 | 0 | 0 | 9 | Low Risk | | Included |  |
| Gosselin et al., 2021 | 1 | 0 | 1 | 0 | 0 | 0 | 1 | 0 | 0 | 0 | 0 | 0 | 1 | 0 | 0 | 0 | 0 | 4 | Medium Risk | | Included |  |
| David Adzrago (2023) | 1 | 0 | 1 | 0 | 1 | 0 | 1 | 0 | 1 | 0 | 1 | 0 | 1 | 0 | 1 | 0 | 0 | 9 | Low Risk | | Included |  |
| Reza Nakhaie (2022). | 1 | 0 | 1 | 0 | 1 | 0 | 1 | 0 | 1 | 0 | 1 | 0 | 1 | 0 | 1 | 0 | 0 | 9 | Low Risk | | Included |  |
| Jerusha Nelson Peterman (2012) | 1 | 0 | 1 | 0 | 1 | 0 | 1 | 0 | 1 | 0 | 1 | 0 | 1 | 0 | 1 | 0 | 0 | 9 | Low Risk | | Included |  |
| Farah Islam (2014). | 1 | 0 | 1 | 0 | 0 | 0 | 1 | 0 | 1 | 0 | 1 | 0 | 1 | 0 | 1 | 0 | 0 | 8 | Low Risk | | Included |  |
| Kari A. Hartwig & Meghan Mason, 2016 | 1 | 0 | 0 | 0 | 1 | 0 | 1 | 0 | 1 | 0 | 1 | 0 | 1 | 0 | 1 | 0 | 0 | 8 | Low Risk | | Included |  |
| Camila A. Pulgar (2015) | 1 | 0 | 1 | 0 | 1 | 0 | 1 | 0 | 1 | 0 | 1 | 0 | 1 | 0 | 1 | 0 | 0 | 9 | Low Risk | | Included |  |
| Myhrvold and Milada (2017) | 1 | 0 | 0 | 0 | 0 | 0 | 1 | 0 | 1 | 0 | 1 | 0 | 1 | 0 | 1 | 0 | 0 | 7 | Low Risk | | Included |  |
| Islam, F. (2018) | 1 | 0 | 1 | 0 | 0 | 0 | 1 | 0 | 1 | 0 | 1 | 0 | 1 | 0 | 1 | 0 | 0 | 8 | Low Risk | | Included |  |
| Andersson (2018) | 1 | 0 | 0 | 0 | 1 | 0 | 1 | 0 | 1 | 0 | 1 | 0 | 1 | 0 | 1 | 0 | 0 | 8 | Low Risk | | Included |  |
| Myhrvold and Milada (2019) | 1 | 0 | 0 | 0 | 0 | 0 | 1 | 0 | 1 | 0 | 1 | 0 | 1 | 0 | 1 | 0 | 0 | 7 | Low Risk | | Included |  |
|  |  |  |  |  |  |  |  |  |  |  |  |  |  |  |  |  |  |  |  | |  |  |
| Attal (2020) | 1 | 0 | 0 | 0 | 0 | 0 | 1 | 0 | 1 | 0 | 1 | 0 | 1 | 0 | 1 | 0 | 0 | 7 | Low Risk | | Included |  |
| Li (2022). | 1 | 0 | 1 | 0 | 1 | 0 | 1 | 0 | 1 | 0 | 1 | 0 | 1 | 0 | 1 | 0 | 0 | 9 | Low Risk | | Included |  |
| Scarlet et al. (2021). | 1 | 0 | 1 | 0 | 1 | 0 | 1 | 0 | 1 | 0 | 1 | 0 | 1 | 0 | 1 | 0 | 0 | 9 | Low Risk | | Included |  |
| Kamelkova (2023) | 1 | 0 | 1 | 0 | 1 | 0 | 1 | 0 | 1 | 0 | 1 | 0 | 1 | 0 | 1 | 0 | 0 | 9 | Low Risk | | Included |  |
|  |  |  |  |  |  |  |  |  |  |  |  |  |  |  |  |  |  |  |  | |  |  |
|  |  |  |  |  |  |  |  |  |  |  |  |  |  |  |  |  |  |  |  | |  |  |
