# Supplementary material for: Food insecurity and mental health among migrants and refugees in high-income countries: Systematic review and meta-analyses
Source: PLoS One. 2026 Feb 18;21(2):e0342128. doi: 10.1371/journal.pone.0342128 (PMC12915952; doi:10.1371/journal.pone.0342128)
Supplement: S1 Fig — (DOCX) [file pone.0342128.s006.docx]

**
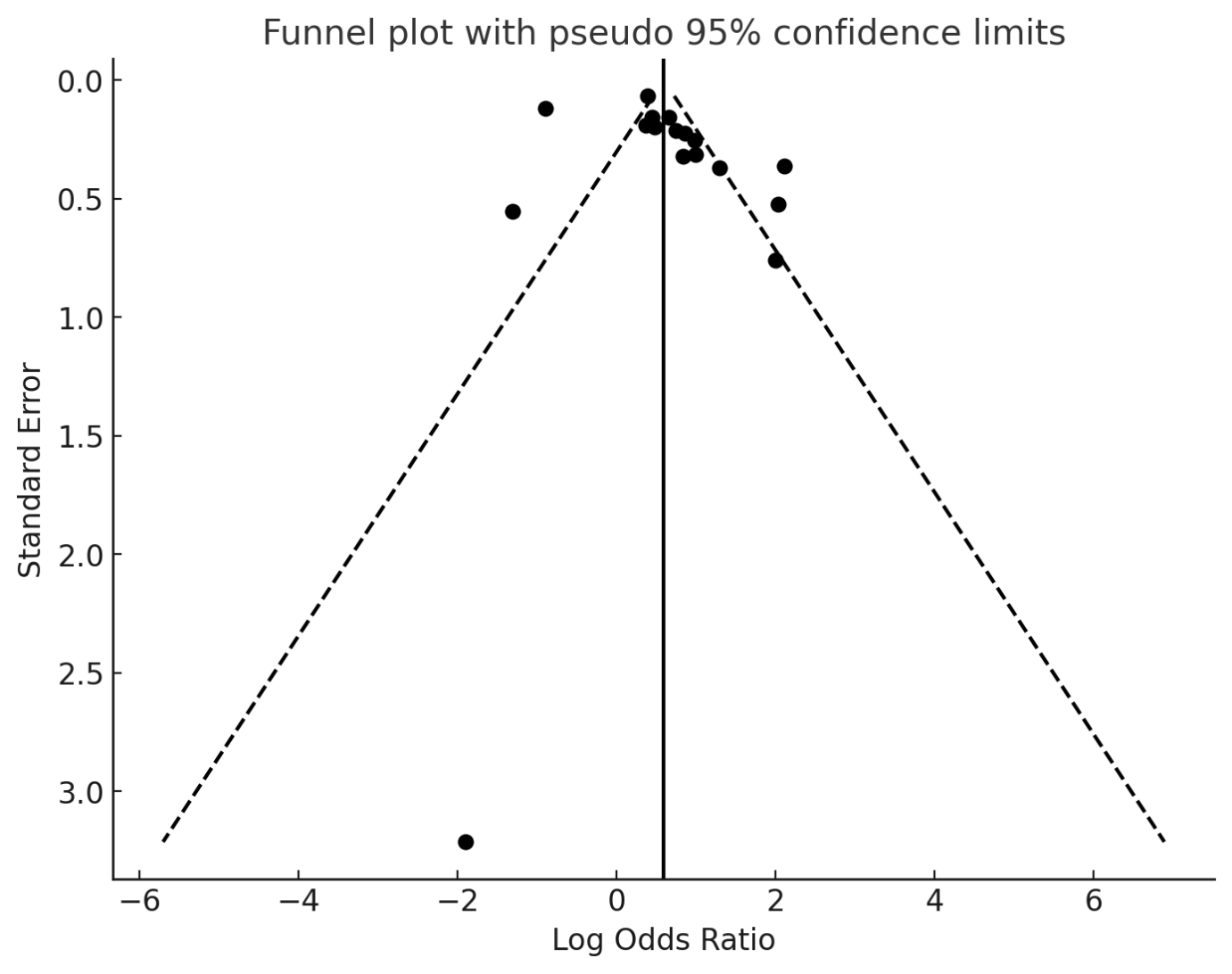
**

S1 Figure. Depression funnel plot 1. Association between FI and Depression

In this funnel plot, the vertical axis indicates the standard error, showing how precise each study's results are, while the horizontal axis represents the log odds ratios, which measure the strength of the relationship between food insecurity and depression. Usually, you'd expect larger, more accurate studies to group near the top, with smaller, less precise studies spreading out towards the bottom, giving the funnel its typical shape. However, in this plot, there’s a clear uneven distribution, especially noticeable on the lower left side. This suggests smaller studies with less significant or negative findings might be missing or unpublished, hinting at potential publication bias. Thus, it's important to interpret the pooled findings carefully, keeping in mind this possibility of missing data.
