# Supplementary material for: Food insecurity and mental health among migrants and refugees in high-income countries: Systematic review and meta-analyses
Source: PLoS One. 2026 Feb 18;21(2):e0342128. doi: 10.1371/journal.pone.0342128 (PMC12915952; doi:10.1371/journal.pone.0342128)
Supplement: S2 Fig — (DOCX) [file pone.0342128.s007.docx]

**
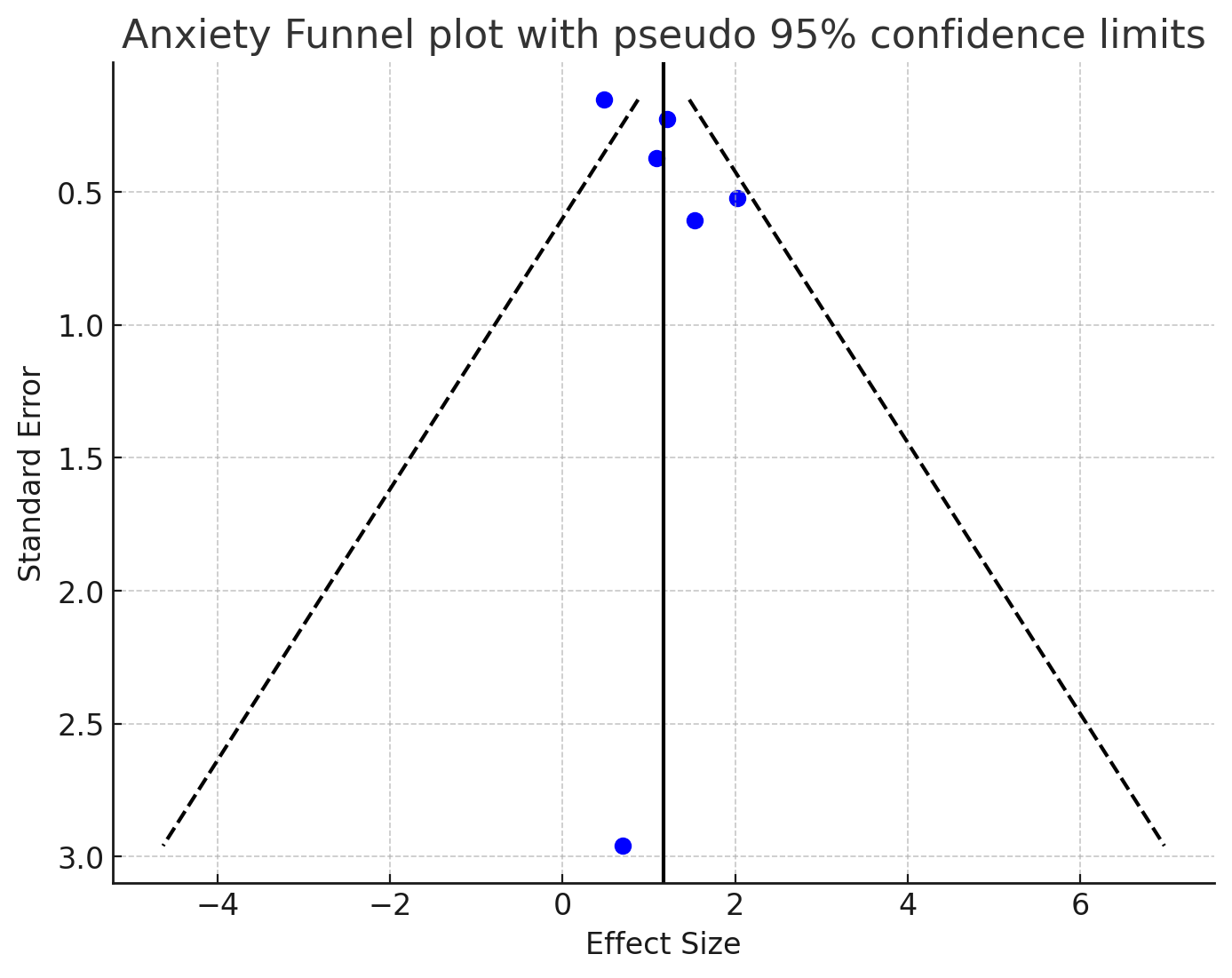
**

S2 Figure. Anxiety funnel plot 2. Association between FI and Anxiety

In this anxiety funnel plot, most studies cluster near the pooled effect line; however, notable asymmetry is visible, particularly with one study positioned far to the lower left. This asymmetry suggests potential publication bias, indicating smaller studies
