# Supplementary material for: Food insecurity and mental health among migrants and refugees in high-income countries: Systematic review and meta-analyses
Source: PLoS One. 2026 Feb 18;21(2):e0342128. doi: 10.1371/journal.pone.0342128 (PMC12915952; doi:10.1371/journal.pone.0342128)
Supplement: Attachment — Submitted filename: Peer Review Report_PONE-D-25-37960.pdf [file pone.0342128.s008.pdf]

Peer Review Report-PONE-D-25-37690

Title: Food Insecurity and Mental Health Among Migrants and Refugees in High-Income Countries: Systematic review and meta-analyses

Original Submission
1.1.Recommendation

Minor revision

2. Comments to Authors:

Ms.Ref No: **PONE-D-25-37960**

Title: Food Insecurity and Mental Health Among Migrants and Refugees in High-Income Countries: Systematic review and meta-analyses

Authors: Resom Berhe[1,2], Amit Arora[2,3,4,5,6], Kanchana Ekanayake[7], Kingsley E. Agho[2,3,8]

Overview and general recommendation:

Overall, the manuscript is well written, technically sound, and presented in an intelligible fashion in standard English.

The impact of food insecurity on the mental health of refugees and migrants in high-income countries is a vital health issue that has not received adequate attention and has thus impacted the implementation of targeted interventions to mitigate the suffering of these vulnerable populations.

The statistical analysis has been performed appropriately and rigorously. The data support the conclusions drawn in the study.

This study has clearly shown the strong association between food insecurity and mental health and provides evidence for targeted interventions in high-income countries for refugees and migrants.

However, there are a few comments I want the authors to address. These are indicated below.

2.1. Comments

1. Indicate the year you are comparing to the number of food-insecure people globally to the numbers in 2020. Line 78 under "Introduction"
2. Correct the word "confidence" in line 270 with the right term "Covidence" software.
