# Supplementary material for: Food insecurity and mental health among migrants and refugees in high-income countries: Systematic review and meta-analyses
Source: PLoS One. 2026 Feb 18;21(2):e0342128. doi: 10.1371/journal.pone.0342128 (PMC12915952; doi:10.1371/journal.pone.0342128)
Supplement: Attachment — Submitted filename: Review report 23.docx [file pone.0342128.s009.docx]

**Peer Review Report**

Peer Review report of the manuscript titled **″ Food Insecurity and Mental Health Among Migrants and Refugees in 1 High-Income Countries: Systematic review and meta-analyses** **″**

The authors effectively highlight critical research questions, appropriately use the statistical test, and thoroughly examine the outcomes. Here are my comments and suggestions for improvement:

**1. Title**

- The title clearly reflects the study goal, systematic review, and meta-analysis.

**2. Abstract**

- The abstract is structured, aligns with the manuscript, and the keywords are informative for readers.

**3. Introduction**

**Research Question & Objectives**

- The introduction effectively describes the background and the research problem.
- The study rationale is clearly identified.
- The research question is clear and focused.
- The study objective clearly describes PICO elements (Population, Intervention/Exposure, Comparator, Outcomes)

**4. Methods**

**Protocol & Registration**

- The review protocol was registered and reported in PROSPERO before the review was conducted.

**Search Strategy**

- A comprehensive search was done across databases; however, the grey literature was not considered.

**Eligibility Criteria**

- Eligibility criteria are clearly defined; nevertheless, study designs are not clearly explained.

**Study Selection Process**

- The study selection process was clearly explained, and three independent reviewers conducted screening.
- The PRISMA flow diagram was included.
- The excluded studies and the reasons for exclusion were clearly documented.

**Data Extraction**

- Data extraction was performed in duplication (by two independent reviewers).

**Risk of Bias / Quality Assessment**

- The quality assessment was done using a suitable quality appraisal approach (Joanna Briggs Institute (JBI) Critical Appraisal Checklist for Analytical Cross-Sectional Studies).

**Data Synthesis**

- An appropriate model of meta-analysis was conducted to examine the statistical combination of results using odds ratios and confidence intervals.
- The heterogeneity among studies was assessed using Cochran’s Q test.
- Risk of bias was clearly assessed.
- Appropriate visual and statistical methods were explained and used to evaluate publication bias (funnel plots, Begg's test, and Egger's test).

**5. Results**

**Results Reporting**

- The characteristics of the included studies are clearly illustrated in the table and text.
- The study outcome was clearly discussed, consistent with the study protocol.
- Sources of funding of included studies in the review are not clearly explained.
- The risk of bias in individual studies is not clearly explained.

**6. Discussion**

**Discussion & Interpretation**

- The findings were interpreted appropriately.
- The strengths and limitations were clearly addressed.
- The conclusion was not supported by evidence.

**Transparency and Reproducibility**

- Data and supplementary materials for reproducibility were provided.

**Writing Quality and Organization**

- The manuscript clearly stated that it followed PRISMA guidelines

**Ethical and Funding Statements**

- There was a clear address for no report for potential conflicts of interest.
- The funding sources were clearly stated.
- **Comments to editors**

I recommend publishing after improvements have been made.

*Best regards*
