# Supplementary material for: Food insecurity and mental health among migrants and refugees in high-income countries: Systematic review and meta-analyses
Source: PLoS One. 2026 Feb 18;21(2):e0342128. doi: 10.1371/journal.pone.0342128 (PMC12915952; doi:10.1371/journal.pone.0342128)
Supplement: Attachment — Submitted filename: Response to Reviewers 10 01 2026 PLOS One.docx [file pone.0342128.s010.docx]

**Response to Reviewers**

Manuscript: “Food Insecurity and Mental Health Among Migrants and Refugees in High-Income Countries: Systematic Review and Meta-Analyses”

Journal: PLOS ONE

Corresponding author: Resom Berhe

Dear Academic Editor and Reviewers,

Thank you for the careful review of our manuscript and for the clear, practical guidance on how to strengthen it. We have revised the manuscript to address each point raised by the editor and reviewers. In brief, we: (i) aligned headings and terminology for consistency, (ii) corrected and replaced the PRISMA flow diagram, (iii) improved figure readability and standardised figure labelling throughout, (iv) clarified eligibility criteria (including study designs) and noted the absence of a systematic grey literature search, (v) expanded the narrative around quality/risk of bias and funding reporting in included studies, and (vi) tightened the opening of the Discussion and tempered causal language in the Conclusions. All edits are highlighted in the marked-up version of the manuscript.

**A. Journal requirements and formatting checks**

**1. PLOS ONE style and file naming**

Response: We reviewed the manuscript against the PLOS ONE formatting samples and adjusted section order and labelling accordingly. The revised files are provided with the requested names.

**2. Funding information placement**

Response: Thank you for this observation; we have removed all funding-related text from the manuscript. Funding details are now provided only via the online submission form, Funding Statement.

**3. Abstract location**

Response: Thank you for this. We have ensured that the Abstract appears immediately after the title page.

**4. Reference to Table 3**

Response: Thank you for pointing this out. We have added an explicit in-text reference to Table 3 in the Sensitivity Analysis section (See line 531, Page 24).

**5. Supporting Information captions**

Response: Thank you very much for raising this point. We have added a “Supporting Information” section at the end of the manuscript, with captions for the supporting files and corresponding in-text citations where appropriate (e.g., for the PRISMA checklist and the full search strategies).

**6. Recommended citations**

Response: We reviewed the suggested citations for relevance and incorporated them where they strengthened the background or interpretation; where they were not directly relevant, we did not add them.

**7. Reference list accuracy and retractions**

Response: Thank you for pointing this out, and we have reviewed the reference list for completeness and consistency. No retracted articles are knowingly cited; if any are identified during final checks, we will either replace them with current evidence or explicitly note the retraction status and rationale in-text.

**B. Additional suggestions from the Academic Editor**

**Editor comment 1: Line 300: Section 2.6 – change “assessment” to “Assessment.”**

Response: Thank you for this. We have updated it to “Quality Assessment” for consistent capitalization (see Line Number 305, Page 11).

**Editor comment 2: Lines 391–411: PRISMA diagram contained “0£” values**

Response: Thank you. We have replaced the PRISMA flow diagram with a corrected version and removed the erroneous values (See line 395, Page 14).

**Editor comment 3: PRISMA on a single page; boxes connected; unclear sentence in second-to-last box**

Response: Thank you for this suggestion. The PRISMA diagram has been redrawn and incorporated as a single-page figure with clearly connected boxes and more precise wording in the relevant sections (see line 395, page 14).

**Editor comment 4: Line 425: Section 3.1 – change “study” to “Study.”**

Response: Thank you for the suggestion. The section heading has been updated to “Characteristics of the Study” to ensure consistency (line 397, page 15).

**Editor comment 5: Table 2: Insert a space after “2.”; standardise heading capitalisation**

Response: Caption spacing and heading capitalisation were standardised for Table 2 (line 441, Page 17).

**Editor comment 6: Table 2: Define all abbreviations and present them in italic font**

Response: A consolidated list of abbreviations was added immediately below Table 2 in italics (Line Number 443-447, Page 18).

**Editor comment 7: Figure 3: Improve clarity and readability**

Response: Figure 3 was replaced with a higher-resolution version, and its caption was added in the requested format (Panels A–D) (see line 482, page 21).

**Editor comment 8: Standardise usage of “Figure” (not “Fig”)**

Response: All in-text references and figure captions were standardised to “Figure” throughout.

**Editor comment 9: Figure 4: Remove repeated title**

Response: We separated the caption from the embedded figure text and retained a single, consistent caption (“Figure 4…”) (See line number 490, Page 21).

**Editor comment 10: Add narrative transitions between Figures 4–5 and Figures 6–7**

Response: We added brief transition sentences in the Results to guide the reader between the primary pooled analyses and the subgroup- and gender-specific figures.

**Editor comment 11: After Figure 7: consistent capitalisation for “Sensitivity Analysis” and “Publication Bias.”**

Response: Heading updated to “Sensitivity Analysis and Publication Bias” to match manuscript style (Line Number 528, Page 24).

**Editor comment 12: Discussion opening paragraph: focus and outline topics**

Response: The opening paragraph of the Discussion was rewritten to clearly preview the key themes addressed (pooled findings, mechanisms/subgroups, limitations, and implications) (See line number 557, Page 25).

**C. Response to Reviewer #1**

**Reviewer #1 comment 1: Indicate the year for the global food insecurity estimate compared with 2020**

Response: Thank you for the comment. We have added the year (2023) to the sentence reporting the “>345 million” estimate, while retaining the comparison to 2020 (line 77, page 3).

**Reviewer #1 comment 2: Correct “confidence” to “Covidence” (software)**

Response: Thank you for the comment. Corrected to “Covidence software” in the Study Selection description (See line number 276, Page 10).

**D. Response to Reviewer #2**

**Reviewer #2 comment 1: Grey literature was not considered**

Response: We clarified in the Methods that a systematic grey literature search was not undertaken and noted this as a limitation that may affect the capture of unpublished evidence ( Line numbers 196-199, Page 7).

**Reviewer #2 comment 2: Study designs not clearly explained**

Response: We clarified the eligible study designs in the Eligibility Criteria (Observational study, including ecological, cross-sectional, case-control, and cohort studies) (line 166, Page 6).

**Reviewer #2 comment 3: Sources of funding of included studies are not clearly explained**

Response: We added text noting inconsistent reporting of funding across the included studies and clarified how funding information was handled during extraction and reporting (See lines 405-408, Page 15).

**Reviewer #2 comment 4: Risk of bias/quality findings not clearly explained**

Response: We expanded the Results (Quality Assessment) narrative to summarise common limitations identified in the JBI appraisal, including the predominance of cross-sectional designs, reliance on self-reported measures, and variation in adjustment for key confounders. We also direct readers to Table 2 for study-level appraisal details (Line number-433-455, Page-16-19).

**Reviewer #2 comment 5: Conclusion not supported by evidence**

Response: We revised the Abstract and main Conclusions to avoid causal language and to align statements closely with the observational evidence base (For Abstract-Line number-49-54, Page-2; for conclusion-line number 664-673, page-29 ).

**We appreciate the opportunity to revise our work and hope the changes meet the journal’s requirements and the reviewers’ expectations.**

Sincerely,

Resom Berhe, PhD student
